# Unusual adult-onset cardiac rhabdomyomas in tuberous sclerosis complex: a case report

**DOI:** 10.3389/fmed.2024.1386089

**Published:** 2024-07-31

**Authors:** H. A. Nati-Castillo, Juan Manuel Quintero, Oswaldo Aguilar Molina, Marlon Arias-Intriago, Fernando P. Melgar Muñoz, Juan S. Izquierdo-Condoy

**Affiliations:** ^1^Interinstitutional Internal Medicine Group (GIMI 1), Department of Internal Medicine, Universidad Libre, Cali, Colombia; ^2^Department of Internal Medicine, Universidad del Valle, Cali, Colombia; ^3^Department of Cardiology, Hospital Universitario del Valle, Cali, Colombia; ^4^One Health Research Group, Universidad de las Américas, Quito, Ecuador

**Keywords:** chest pain, cardiac rhabdomyoma, tuberous sclerosis, echocardiography, adult onset

## Abstract

**Background:**

Tuberous sclerosis complex is a genetic neurocutaneous autosomal dominant syndrome, characterized by the development of multiple benign tumors (hamartomas) affecting various systems. Heart-benign tumors that result from the complex are called cardiac rhabdomyomas. Unlike hamartomas that occur in other organs, cardiac rhabdomyomas are most prevalent in infants and very young children with tuberous sclerosis complex. We present a case of a young adult with tuberous sclerosis who had an unusually late diagnosis of cardiac rhabdomyomas.

**Case report:**

A 22-year-old male patient of Afro-descendant, diagnosed with tuberous sclerosis complex in childhood, presented with refractory epilepsy and was treated only with lacosamide. The patient came to medical consultation due to a recent history of episodic, persistent chest pain in the sternal region, associated with physical effort. Echocardiography revealed a non-dilated left ventricle, with several rounded masses of high echogenicity without pedicles at the apical level, the largest measuring 14 × 11 mm, consistent with cardiac rhabdomyomas.

**Conclusion:**

Cardiac rhabdomyomas rarely develop in adulthood for individuals with tuberous sclerosis. These late-onset cases can exhibit various symptoms, from simple to complex presentations. Regular clinical checkups are essential for adults with tuberous sclerosis complex.

## 1 Introduction

Tuberous sclerosis complex is a rare genetic neurocutaneous autosomal dominant syndrome in humans, caused by mutations in TSC1 or TSC2 gene. It is characterized by the growth of benign tumors (hamartomas) in multiple locations, including organs such as the skin, brain, kidneys, heart, and lungs. Heart benign tumors resulting from tuberous sclerosis complex are called cardiac rhabdomyomas, composed of normal heart tissue (rhabdo) that grows in a disorganized mass (myoma) (1, 2). Unlike hamartomas that occur in other organs, cardiac rhabdomyomas are most prevalent in infants and very young children with tuberous sclerosis complex. In fact, these are the only lesions that regress over time in tuberous sclerosis, in some cases disappearing entirely (3, 4). In rare cases, large or several cardiac rhabdomyomas that grow on or near the heart valves can disrupt heart rhythm or blood flow, causing angina symptoms and possibly death (5).

Clinically, up to 64% of patients present the syndrome asymptomatically, and a smaller percentage (approximately 35%) present symptoms expressed by arrhythmias and Wolff–Parkinson–White syndrome. The estimated mortality associated with cardiac rhabdomyomas due to cardiac complications has been estimated to be close to 2% in cohorts of patients (5–8). Abnormalities within the echocardiogram have been reported in up to 39%, with focal areas of intramyocardial hyperechogenicity being the most prevalent (9).

We present a case of tuberous sclerosis complex accompanied by cardiac rhabdomyomas with symptomatic expression in adulthood.

## 2 Case presentation

A 22-year-old male patient of Afro-descendant, diagnosed with tuberous sclerosis complex in childhood, presented with refractory epilepsy and was treated only with 100 mg lacosamide every 12 h. He has an opioid allergy (tramadol). His family history includes a father with obesity and type 2 diabetes mellitus, a mother with high blood pressure, and a maternal grandfather with prostate cancer. He has no family history of tuberous sclerosis. Previous interventions identified unspecified epilepsy on electroencephalogram 8 years ago (2015), with no past surgical interventions.

The patient sought medical consultation for a 1-year history of episodic, persistent chest pain in the sternal region, lasting 1 to 5 min and exclusively associated with physical effort. There is no relief with any specific factor.

**Figure 1:**
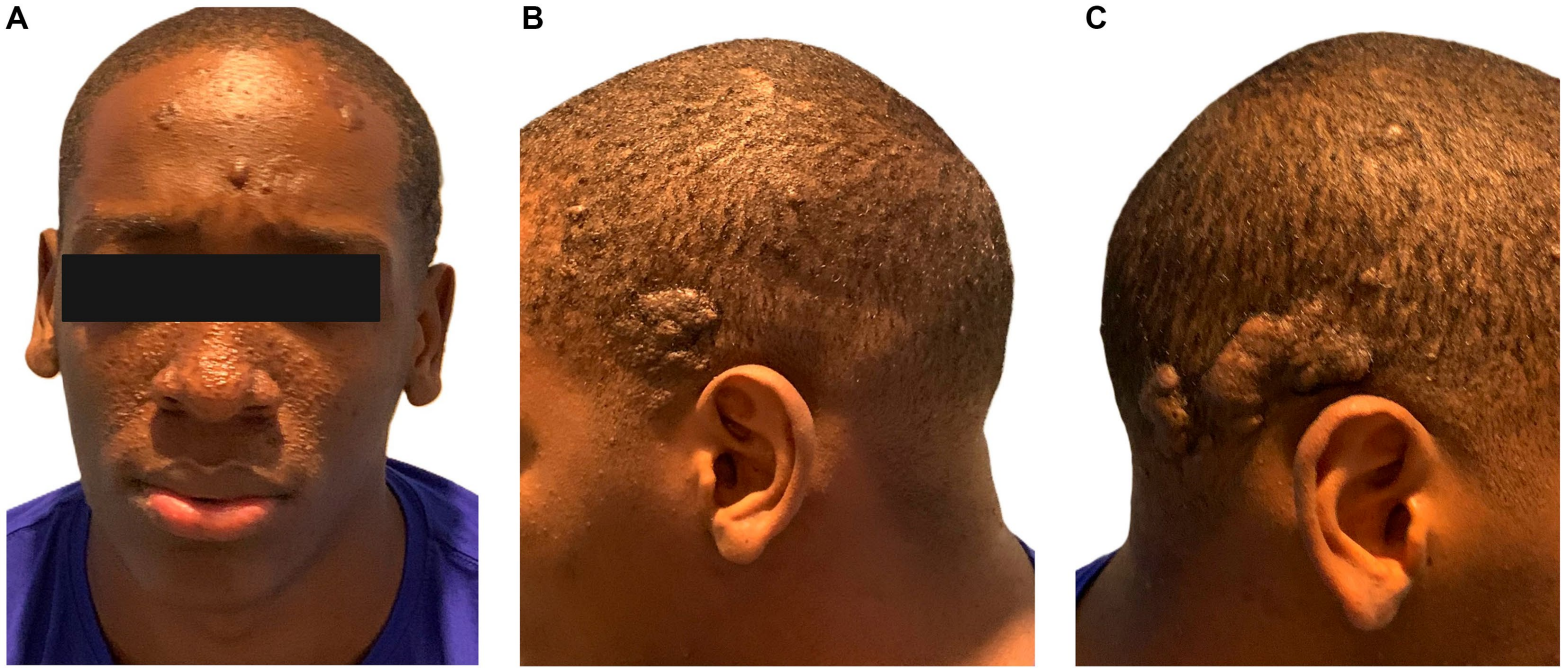
Phenotype of a 22-year-old male patient with tuberous sclerosis. **(A)** Phototype V, at frontal-level euchromatic nodules, soft smooth surface about angiofibroma. In the middle facial third, papules smaller than 5 mm in diameter, smooth euchromatic and violaceous surface, regular distribution on nasal dorsum and bilateral malar region in relation with angiofibromas. **(B)** Hairy skin left temporo-occipital region: exophytic tumor, well-defined irregular borders, smooth surface with convolutions, not ulcerated, size 4 cm by 3 cm. **(C)** Hairy skin right temporo-occipital region: exophytic tumors, well defined, irregular borders, smooth surface with convolutions, not ulcerated, size 5 cm by 3 cm and 2.5 cm by 1.5 cm, respectively.

On physical examination, the examination of the left eye fundus revealed slightly elevated yellowish-white xanthochromic lesions at the end of the upper temporal arch, suggestive of and compatible with retinal hamartomas. The abdominal cavity showed no alterations. A cardiac examination revealed a systolic murmur in the mitral and aortic focus, grade III/IV. The respiratory system showed no abnormalities. The skin and integumentary system exhibited multiple elevated lesions without inflammatory changes, which were not painful and compatible with angiofibromas located on the face and head (Figure 1).

**Figure 2:**
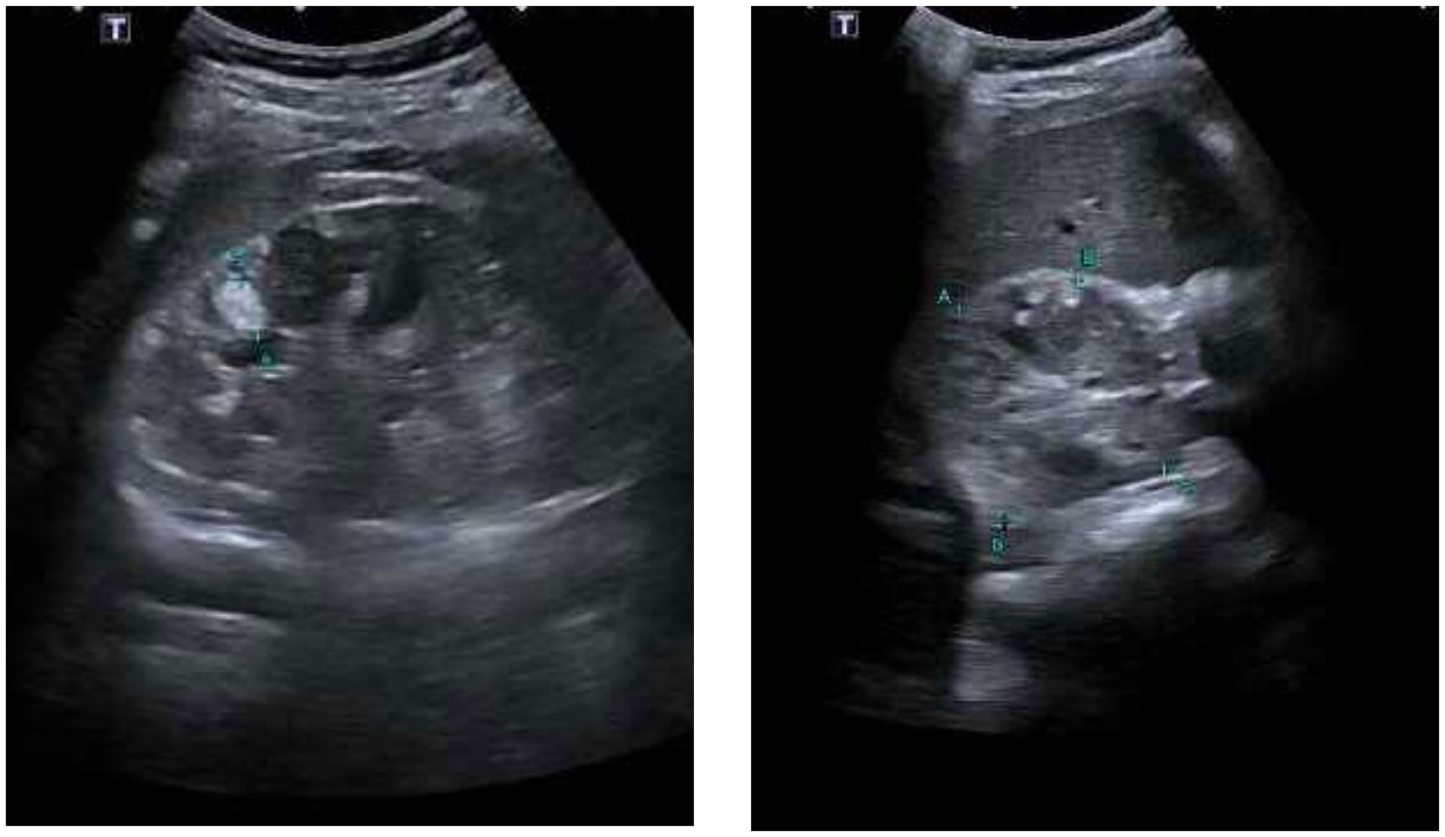
Bilateral renal ultrasound. Parenchyma displays multiple small rounded echogenic images distributed throughout, with sizes ranging between 12.5 mm, 10 mm, 7 mm, 5 mm, and 4 mm.

**Figure 3:**
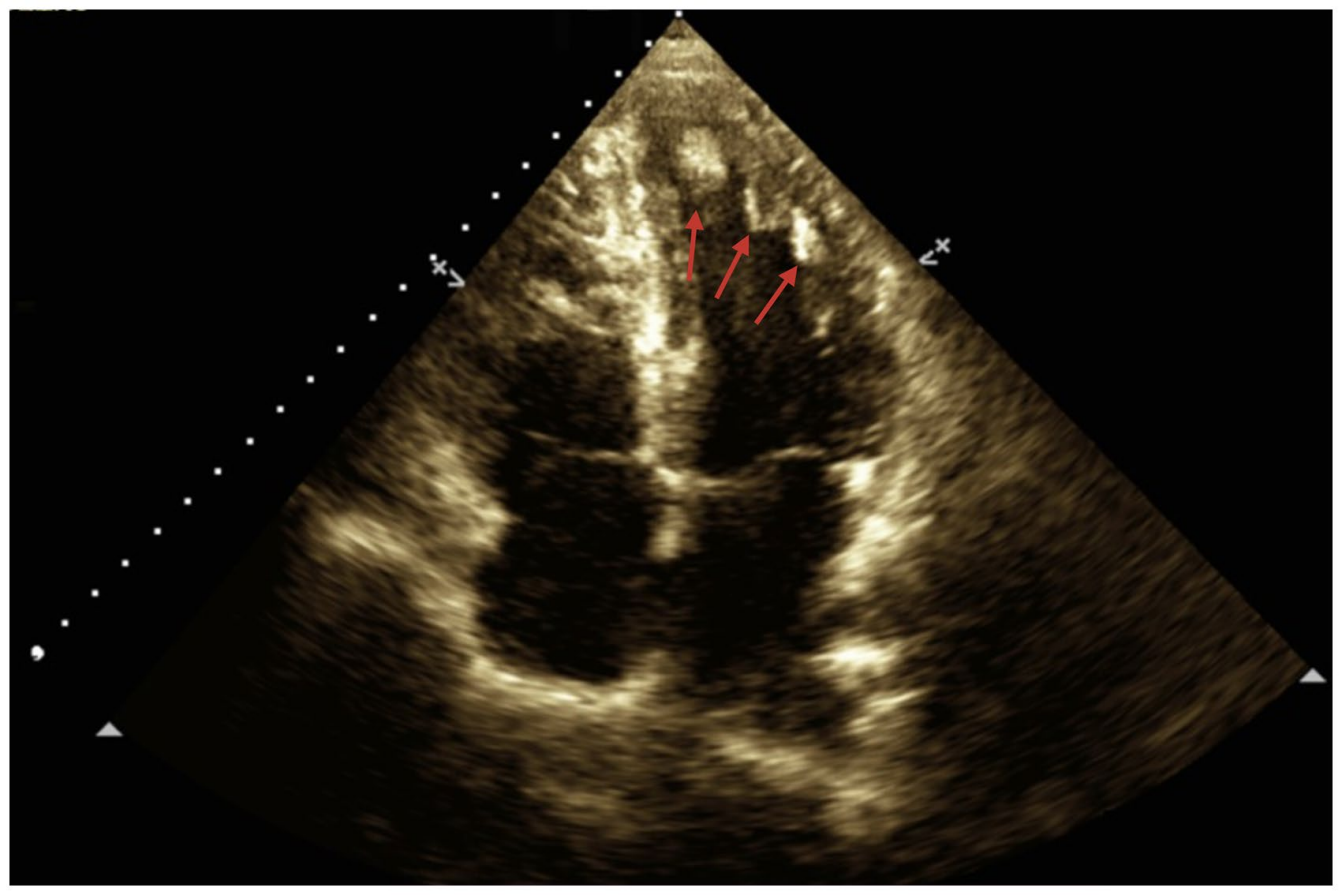
Two-dimensional echocardiography of the patient, showing an apical four-chamber view. The red arrows indicate several homogeneous masses with well-defined borders, retractile and without pedicles, affecting the entire interventricular septum and apical region. The largest mass, located closer to the interventricular septum, measures approximately 14 mm × 11 mm.

Given the patient’s condition, the initial electrocardiogram showed no alterations. Consequently, diagnostic studies, including a brain MRI a few weeks later, were performed. The MRI revealed small bilateral subependymal lesions, the largest measuring 1.6 × 1.6 cm on the lateral wall of the occipital extension of the right lateral ventricle. Additionally, approximately 4 months later, renal ultrasound and echocardiogram were performed. The renal ultrasound showed bilateral renal involvement with multiple rounded masses of variable size (ranging between 12.5 mm and 4 mm) at the parenchymal level, characteristic of angiomyolipomas (Figure 2). Echocardiography revealed a non-dilated left ventricle with several rounded masses of high echogenicity without pedicles at the apical level, the largest measuring 14 × 11 mm. These masses were retractile, with mobility toward the ventricular wall, consistent with cardiac rhabdomyomas (Figure 3). No contractility disorders were observed, and the ejection fraction was 60% (Simpson’s biplane method). The atria, valves, and the right ventricle showed no abnormalities.

At the diagnosis of a cardiac rhabdomyoma in the left ventricle, the patient received symptomatic management for the chest precordial pain using neuromodulators (Pregabalin). Cardiological surveillance was initiated, which, after 4 months (March 2024) of management, has shown improvement in pain without other significant symptoms.

## 3 Discussion

Tuberous sclerosis or Bourneville’s disease of autosomal dominant genetic origin is a very rare syndrome (5–7/10,000 live births) (10). The spectrum of clinical manifestations of the syndrome includes neurological manifestations, such as autism spectrum disorders, cortical tuberomas, epilepsy, and intellectual disability, with the latter being the most common (11, 12). Main cutaneous manifestations include facial angiofibromas (formerly sebaceous adenomas), periungual fibromas, ash-leaf spots, and hypochromic macules (up to 80%). Cutaneous clinical manifestations are essential for reliably approximating the clinical diagnosis of the disease (11).

Cardiac manifestations encompass cardiac rhabdomyomas, which are benign tumors considered the most common since perinatal diagnosis (60%). They can be detected ultrasonographically even in the prenatal period (between 20 and 30 weeks), with the most common location being in the ventricles (70%) (13). These tumors usually behave asymptomatically; however, they can interfere with valvular function, alter the ventricular inflow or outflow tract, generate anomalies in the cardiac conduction system, or occlude the adequate irrigation of the coronary system. This can lead to myocardial ischemic events, triggering heart failure and arrhythmias in up to 43.3% (13, 14).

In this case, although the patient shares characteristics similar to those reported in the literature, such as the clinical diagnosis of tuberous sclerosis based on common manifestations, including neurological (epilepsy), cutaneous (angiofibromas), and ocular (hamartomas) during childhood, a distinctive aspect of this report is that the key clinical manifestation for discovering cardiac rhabdomyomas occurred in young adulthood (21 years). This manifestation presented as chest pain of prolonged duration (1 year), which, along with findings from the initial examination and the patient’s history, led to a transthoracic echocardiographic study. This study revealed multiple lesions, leading to the diagnosis of cardiac rhabdomyomas, without evidence of damage to cardiac conduction, highlighting a very unusual presentation. It is important to note that the nature of the case reflects the patient’s environment with limited resources, living in a rural area with difficult access to healthcare, which contributed to the prolonged progression and delay in obtaining a definitive diagnosis.

This case is of interest because, according to the recent review by Frudit et al. (13), it could be the second to date of a young adult with tuberous sclerosis that had a late diagnosis of cardiac rhabdomyomas, characterized only by chest pain. The only previous report was by Enbergs et al. (15) in 1996 on a 23-year-old patient accompanied by ventricular tachycardia.

Although a relationship between ethnicity and the syndrome or its accompanying features has not been established, apparently, presentation in individuals of African descent is rare (16, 17). This case broadens the phenotypic picture of tuberous sclerosis associated with cardiac rhabdomyomas by presenting in an individual of African descent ethnicity. It proposes considering ethnicity as a variable to study in future patients’ reports with tuberous sclerosis complex with rare features.

Despite not all patients with tuberous sclerosis presenting with cardiac rhabdomyomas, this case highlights the importance of active cardiac screening for these patients during the disease, given the potential complications even in adulthood. In this context, the standard of choice is cardiac echocardiography with a complete Doppler study, which shows well-circumscribed, nodular, multiple masses in the myocardium, which usually project into the ventricular chamber (14). Their diagnosis can be difficult if they present atypical locations such as the atria or present as solitary tumors (5). Additionally, the clinician may opt for cardiac MRI, which, although not widely available in developing settings like this case, can provide a more accurate description of the masses when the echocardiographic diagnosis is unclear and as a useful adjunct when surgical resolution is planned (14). The follow-up of this entity is usually based on growth monitoring with echocardiography and electrocardiograms every 3 to 5 years to assess possible conduction disorders and risks of disease progression such as heart failure (18).

## 4 Conclusion

The late onset of cardiac rhabdomyomas in adulthood among patients with tuberous sclerosis is particularly rare and may manifest symptomatically with a variety of presentations, ranging from simple to complex. Regular clinical inspections should be conducted for individuals with tuberous sclerosis complex, even in adulthood, to monitor the emergence of benign cardiac masses.

## Data availability statement

The original contributions presented in the study are included in the article/supplementary material, further inquiries can be directed to the corresponding author.

## Ethics statement

Written informed consent was obtained directly from the patient. Written informed consent was obtained from the participant/patient(s) for the publication of this case report.

## Author contributions

HN-C: Conceptualization, Data curation, Formal analysis, Investigation, Methodology, Project administration, Resources, Validation, Visualization, Writing – original draft. JQ: Conceptualization, Data curation, Investigation, Methodology, Resources, Validation, Visualization, Writing – original draft. OM: Conceptualization, Data curation, Investigation, Methodology, Resources, Validation, Visualization, Writing – original draft. MA-I: Formal analysis, Investigation, Methodology, Resources, Software, Visualization, Writing – original draft. FM: Formal analysis, Investigation, Methodology, Resources, Software, Visualization, Writing – review & editing. JI-C: Data curation, Funding acquisition, Investigation, Methodology, Project administration, Software, Supervision, Validation, Visualization, Writing – original draft, Writing – review & editing.
